# Highlight selection of radiochemistry and radiopharmacy developments by editorial board

**DOI:** 10.1186/s41181-024-00296-6

**Published:** 2024-09-16

**Authors:** Peter JH. Scott, Ivan Penuelas, Ana Rey, Silvio Aime, Pillai M.R. Ambikalmajan, Ines Farinha Antunes, Frederik Cleeren, Zhaofei Liu, Beverley Ellis, Maryke Kahts, Fany Pricile Ekoume, Ivis F. Chaple, Emerson Bernardes, Martin Behe, Ya-Yao Huang, Renata Mikolajczak, Shozo Furumoto, Amal Elrefaei, Klaus Kopka

**Affiliations:** 1 University of Michigan, Ann Arbor, MI USA; 2 University Clinic of Navarra, Pamplona, Spain; 3 Universidad de la Republica, Montevideo, Uruguay; 4 University of Torino, Torino, Italy; 5 Molecular Group of Companies, Kochi, Kerala India; 6 University Medical Center Groningen, Groningen, Netherlands; 7 Katholieke Universiteit, Leuven, Belgium; 8 Peking University, Beijing, China; 9 Manchester University NHS Foundation Trust, Manchester, UK; 10 Sefako Makgatho Health Sciences University, Ga-Rankuwa, South Africa; 11 University of Yaoundé 1, General Hospital, Yaoundé, Cameroon; 12 Department of Nuclear Engineering, University of Tennessee Knoxville, Knoxville, USA; 13 IPEN, Sao Paulo, Brazil; 14 Paul Scherrer Institute, Villigen, Switzerland; 15 Primo Biotechnology Co. Ltd. Taipei, Taipei, Taiwan; 16 National Centre for Nuclear Research, Radioisotope Centre POLATOM, Otwock, Poland; 17 Research Center for Accelerator and Radioisotope Science, Tohoku University, Sendai, Japan; 18 International Atomic Energy Agency (IAEA) Vienna, Vienna, Austria; 19 Institute of Radiopharmaceutical Cancer Research, Helmholtz-Zentrum Dresden-Rossendorf (HZDR), Faculty of Chemistry and Food Chemistry, School of Science, Technical University Dresden (TUD), Dresden, Germany

**Keywords:** Highlight articles, Radiochemistry, Radiopharmacy, Radiopharmaceutical sciences, Nuclear medicine

## Abstract

**Background:**

The Editorial Board of EJNMMI Radiopharmacy and Chemistry releases a biannual highlight commentary to update the readership on trends in the field of radiopharmaceutical development.

**Main body:**

This selection of highlights provides commentary on 19 different topics selected by each coauthoring Editorial Board member addressing a variety of aspects ranging from novel radiochemistry to first-in-human application of novel radiopharmaceuticals.

**Conclusion:**

Trends in radiochemistry and radiopharmacy are highlighted. Hot topics cover the entire scope of EJNMMI Radiopharmacy and Chemistry, demonstrating the progress in the research field in many aspects.

## Background

Each individual coauthoring member of the Editorial Board has selected to highlight an article that has appeared in the radiochemistry, radiopharmacy and imaging agent literature during the period January-June 2024. The aim of this collaborative initiative is to create a biyearly overview for the readers summarizing the latest trends and hot topics in the field.

## Selected highlight articles

### A hot new method for preparing isotopologues of the trifluoromethyl group!

*By Peter J. H. Scott*

**Fig. 1:**
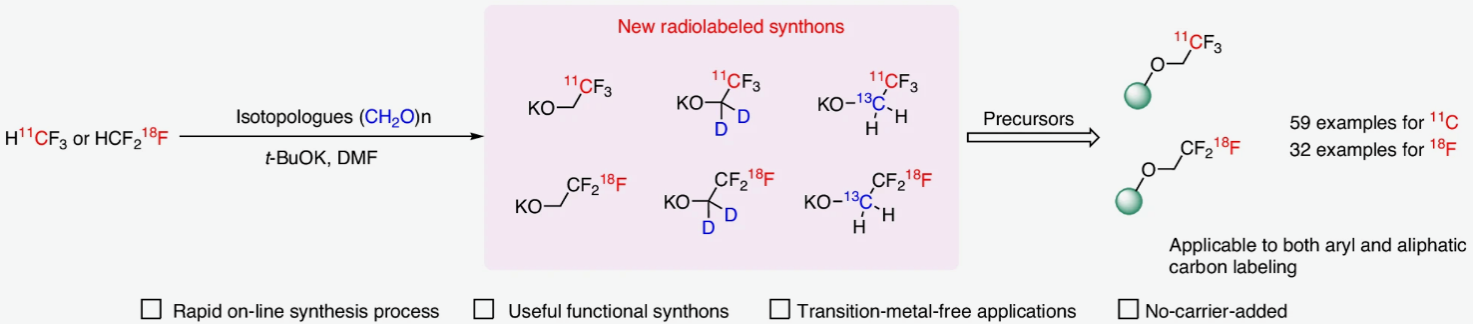
New methodology for rapid and efficient radiotrifluoroethoxylations. Reproduced from (Zhao et al., 2024), under a Creative Commons Attribution 4.0 International License

The trifluoromethyl (CF_3_) group is an important functional group in medicinal chemistry that is frequently incorporated into drugs to improve pharmacokinetic and physicochemical properties, and can be utilized as a bioisostere for a conventional methyl group. There is thus considerable interest in making isotopologues of the trifluoromethyl group, labeled with positron-emitting radionuclides like ^11^C and ^18^F, such that CF_3_-containing drugs can be rapidly adapted for PET imaging applications (Francis and Wuest 2021). The 2,2,2-trifluoroethoxide group is an attractive way of incorporating a CF_3_ group into bioactive molecules owing to its metabolic stability and reasonable lipophilicity. However, existing methods to label 2,2,2-trifluoroethoxides have limitations (not general, radiochemical yields limited by side product formation, low molar activity). To address this issue, Zhao and colleagues have developed an exciting method of preparing ^11^C- and ^18^F-labeled potassium 2,2,2-trifluoroethoxide [^11^C/^18^F]CF_3_CH_2_OK) from the corresponding labeled fluoroform *via* reaction with an aldehyde (Fig. 1) (Zhao et al. 2024). Labeled potassium 2,2,2-trifluoroethoxides were reacted with a variety of aromatic (iodonium salts, iodonium ylides, fluorides), heteroaromatic (halides, nitro, SO_2_Me) and aliphatic (tosylates, halides) precursors under very mild conditions (room temp-60 ^o^C, 1–3 min) to yield ^11^C- and ^18^F-trifluoromethylated products (^11^C: 59 examples, 12–94%; ^18^F: 32 examples, 15–96%; decay-corrected yields based upon fluoroform). The method is tolerant of diverse functional groups, electronics in the case of (hetero)aromatics, and is compatible with complex bioactive and drug like molecules. Lastly, reacting stable (D,^13^C) labeled aldehydes with fluoroform enabled access to dual labeled probes with potential applications in hybrid imaging (Fig. 1). Overall, this new method is quite general and should facilitate radiotracer development around the 2,2,2-trifluoroethoxy group going forward.

### ^177^Lu-labeled manganese-doped mesoporous hydroxyapatite microspheres for enhanced tumor in situ vaccination

*By Ivan Penuelas*

Tumor in situ vaccination (ISV) strategies, which involve the release of tumor antigens through local radiotherapy and intratumoral adjuvant injections, have shown promise in clinical trials. However, achieving a sustainable immune response remains challenging.

**Fig. 2:**
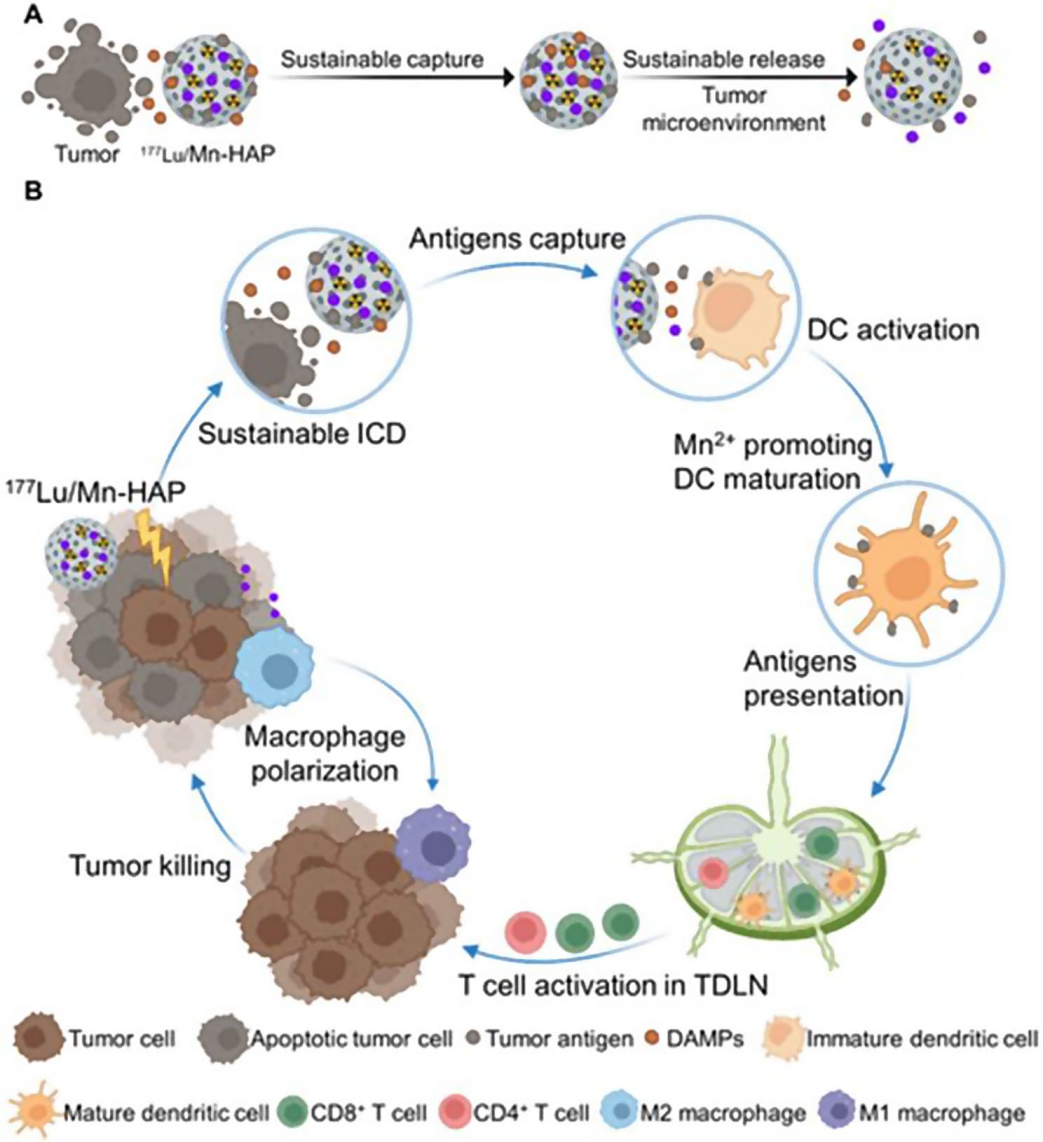
Schematic Illustration of the ISV Fabrication of ^177^Lu/Mn-HAP for Sustainable Antitumor Immune Response. (**A**) Sustained tumor antigen capture and release of ^177^Lu/Mn-HAP. (**B**) Activation of systemic antitumor immune response by ^177^Lu/Mn-HAP. Reprinted from Xu P et al. Radioactive Hydroxyapatite Microspheres Empower Sustainable In Situ Tumor Vaccination. ACS Nano 2024: 18,425–18,443

A recent study introduces an enhanced ISV method using ^177^Lu-labeled manganese-doped mesoporous hydroxyapatite (^177^Lu/Mn-HAP) microspheres that induce immunogenic cell death (ICD) in tumor cells (Xu et al. 2024). Released antigens are captured and gradually released by the mesoporous structure, maintaining high local antigen concentrations (Fig. 2). This process leads to dendritic cell activation and macrophage polarization toward the M1 subtype, enhancing CD8+ T-cell responses and antitumor effects in both primary and distant tumors.

The cyclic GMP-AMP synthase (cGAS) agonist Mn²⁺, used as an immune adjuvant, further amplifies dendritic cell maturation and macrophage polarization, activating the cGAS-stimulator of interferon genes signalling pathway (Wang et al. 2018).

Comprehensive characterization confirmed the successful loading and release of lutetium-177 and Mn²⁺ by the ^177^Lu/Mn-HAP microspheres. Cellular experiments and proteomic analyses demonstrated their ability to induce tumor cell death and capture released antigens, promoting ICD. In vitro studies revealed that ^177^Lu/Mn-HAP stimulates dendritic cells and shifts macrophages toward an M1 polarization state.

In tumor-bearing mice, intratumorally injected ^177^Lu/Mn-HAP achieved a 100% complete remission rate, activating systemic immunity and fostering immune memory, thus preventing tumor recurrence. In vivo SPECT/CT imaging showed stable retention at the tumor site, minimizing radiation damage to surrounding tissues.

By using composite radioactive microparticles, this study highlights a universal and safe ISV strategy capable of inducing potent tumor-specific and sustainable immune responses.

### Development of potential radiopharmaceuticals directed towards multiple molecular targets: a step forward in personalized oncological theranostics?

*By Ana Rey*

Cancer is a complex disease comprising many subtypes with significant molecular differences. Subpopulations of cells with different biological behavior can coexist within a primary tumor and its metastasis, or in tumors of the same histopathological subtype. Furthermore, molecular targets within a tumor may change in different stages of the disease or be affected by the therapy.

Peptide-based radiopharmaceuticals have been very successful in targeting the tumor cells while sparing the normal ones. The traditional approach is to design monovalent radiotracers directed towards only one receptor overexpressed in the specific type of tumor. However, a new paradigm has arisen recently, the multivalent approach in which a heterodimeric radioligand can bind simultaneously to two different molecular targets of the cancer cell. This new concept offers a versatile solution to overcome the heterogeneous nature of tumors and holds promise for personalized care in oncology.

A recent publication presents a thorough review of the state of the art in the design of dual receptor targeting radiopharmaceuticals (Chambers et al. 2024). Many efforts have been dedicated to the combination of RGD peptides directed to the integrin α_V_β_3_ with different peptides (bombesin, alpha-melanocyte-stimulating hormone or somatostatin analogs) and PSMA inhibitors, highlighting the importance of angiogenesis and other processes associated with the extracellular matrix for molecular imaging and targeted therapy. Despite the inherent challenges associated with the synthesis of heterobivalent radioligands the results are promising in terms of enhanced sensitivity and potential to encompass a wider range of patients.

### Improving the in vivo stability of [^52^Mn]Mn(II) complexes with 18-membered macrocyclic chelators for PET imaging

*By Silvio Aime*

**Fig. 3:**
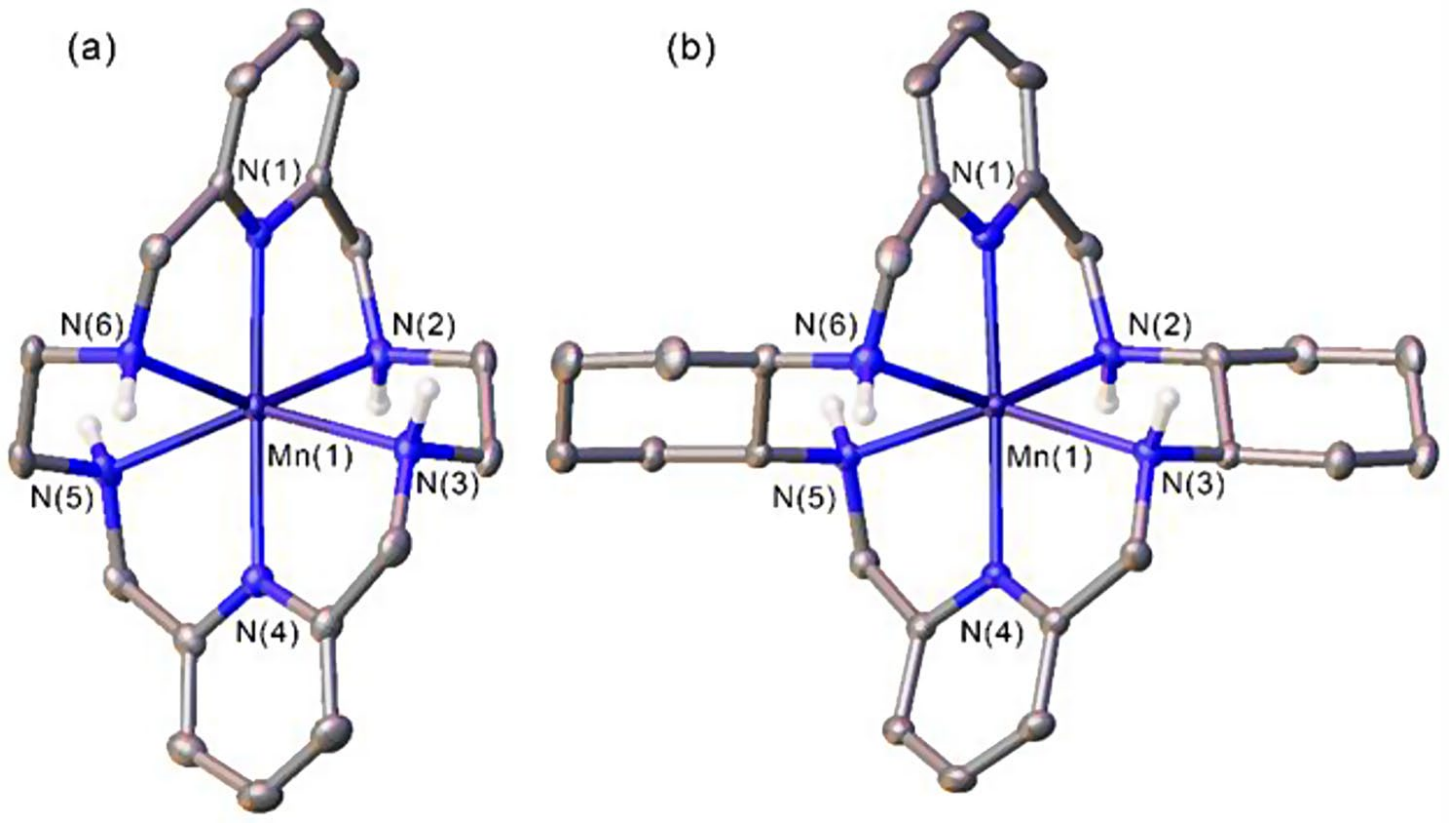
Two Mn(II)-complexes with 18-membered macrocycles PYAN (**a**) and CHXPYAN (**b**)

Manganese is a promising metal for both MRI and PET applications. One of the proposed manganese radioisotopes for PET imaging is manganese-52 (^52^Mn, *t*_1/2_ = 5.6 days), a cyclotron produced, long-lived positron emitter that can be used to obtain high-resolution images several days after injection. Two promising Mn(II)-complexes with 18-membered macrocycles PYAN (a) and CHXPYAN (b) have been investigated for their potential in vivo applications (Harriswangler et al. 2024) (Fig. 3).

The coordination polyhedra around the Mn(II) ions can be described as severely distorted octahedra. The redox properties suggest that these Mn(II)-complexes are resistant to oxidation in biological media. The stability constants of [Mn(CHXPYAN)]^2+^ is slightly higher than that of [Mn(PYAN)]^2+^, but both complexes have higher log*K*_MnL_ values than parent [Mn(18-ane-N_6_)]^2+^ indicating the improved thermodynamic properties of Mn(II) complexes with pyridyl and cyclohexyl units in the backbone. The relaxivity is fairly constant in the pH range of 6 − 9 with values typical of outer-sphere systems. The kinetic inertness has been assessed by following the transmetalation of [Mn(CHXPYAN)]^2+^ and [Mn(PYAN)]^2+^ with Cu^2+^. Mechanism implies the rate determining acid catalyzed dissociation (*k*_1_) of the Mn(II) complexes followed by the fast reaction between the free ligands and the Cu^2+^ ion. The value of *k*_1_ determined for [Mn(CHXPYAN)]^2+^ is 3600 times lower than that determined for [Mn(PYAN)]^2+^, highlighting the beneficial impact of the rigid cyclohexyl units on the kinetic inertness of the Mn(II)-complex. Human and mouse serum stability of [^52^Mn(CHXPYAN)]^2+^ showed a 98% intact complex at 5 days post incubation. In vivo studies were carried out with a comparison to the biodistribution of uncomplexed ^52^MnCl_2_. [^52^Mn(CHXPYAN)]^2+^ showed a different biodistribution profile as compared with that of free ^52^Mn(II) with fast clearance from the liver and kidneys 1.5 h post-injection. Vice versa [^52^Mn(PYAN)]^2+^ showed a similar distribution pattern as compared with that of unchelated ^52^Mn(II) with persistent accumulation of radioactivity in the liver, pancreas, and spleen. In summary this work showed that CHXPYAN is a very promising platform for the development of ^52^Mn(II)-based radiopharmaceuticals. Comparison of the Mn(II) complex of this chelator with that of its more flexible analogue, PYAN, shows that the incorporation of two cyclohexyl units into the 18-membered backbone confers increased thermodynamic stability and kinetic inertness. This work is an important contribution to the development of new bifunctional systems for ^52^Mn(II)-based radiopharmaceuticals.

### Quality control of actinium-225 radiopharmaceuticals: let us isolate the ^221^Fr gamma photons for the measurement

*By Pillai M.R. Ambikalmajan*

Quality control of radiopharmaceuticals labeled with actinium-225 (^225^Ac) is a challenging task due to the presence of multiple radionuclides in its decay chain. All these radionuclides are in secular equilibrium with ^225^Ac and will therefore be present in the sample. To determine the radiochemical purity (RCP), it is essential to account for both the ^225^Ac bound to the targeting molecule through a chosen complexing agent and the unbound (free) ^225^Ac. A reliable method for accurate quality control of ^225^Ac-radiopharmaceuticals was recently described by Hooijman et al. (2024).

In this publication, rapid quality control tests followed by gamma counting are used to estimate the radiochemical yield (RCY) and RCP. There are three gamma emissions in the ^225^Ac decay chain, originating from ^225^Ac, francium-221 (^221^Fr), and bismuth-213 (^213^Bi), respectively. The authors measured gamma photons from ^221^Fr or ^213^Bi, ensuring that the counted activity corresponded to ^225^Ac.

Francium-221 and ^213^Bi are radiometals and are expected to coordinate with the complexing agent in the targeting molecule. Therefore, ^221^Fr- and ^213^Bi-labeled variants will be present alongside the respective ^225^Ac-radiopharmaceutical. Uncomplexed ^225^Ac, ^221^Fr, and ^213^Bi will also be present in the free component. Counting the chromatography strip immediately after development would thus yield inaccurate results.

**Fig. 4:**
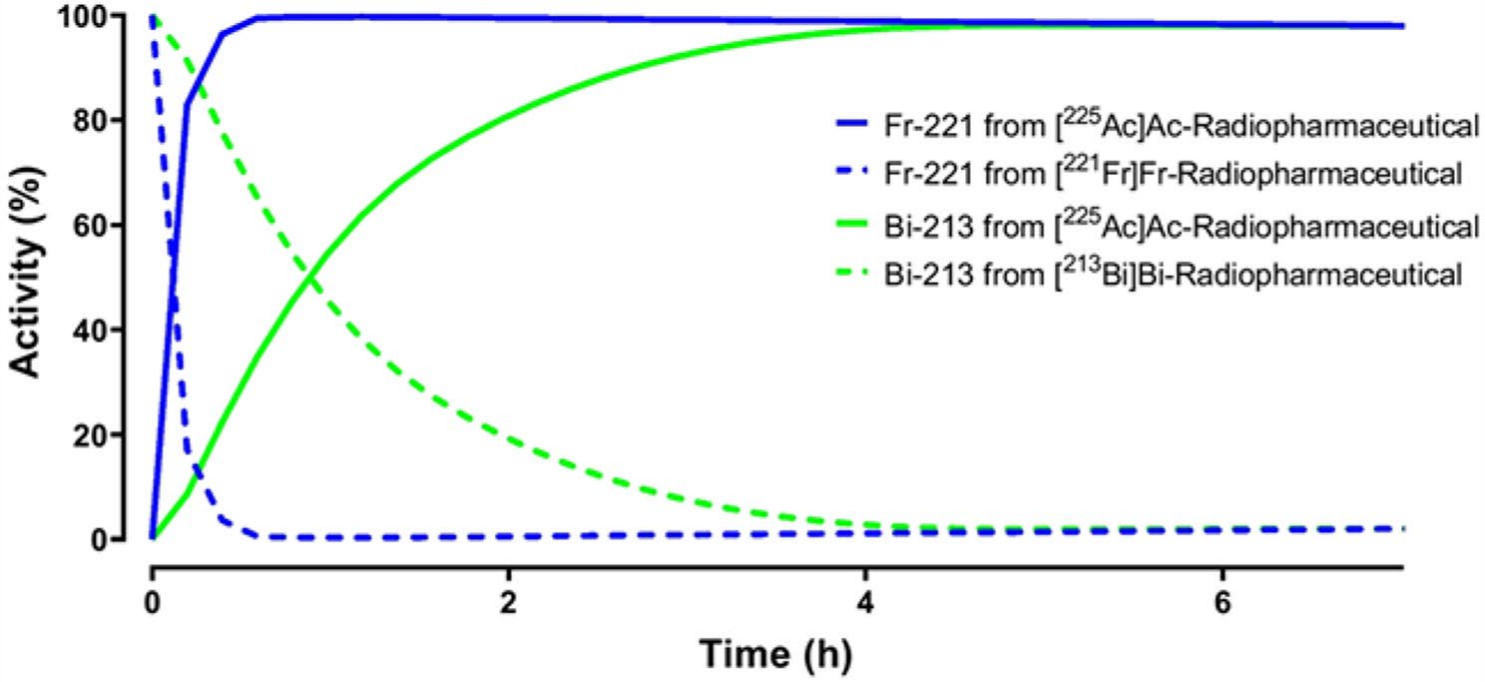
Presence of ^221^Fr and ^213^Bi in different chemical forms after ^225^Ac-labelling of PSMA-I&T resulting in [^225^Ac]Ac-PSMA-I&T. From: Hooijman EL, Radchenko V, Ling SW, Konijnenberg M, Brabander T, Koolen SLW, de Blois E. Implementing ^225^Ac-labelled radiopharmaceuticals: practical considerations and (pre-) clinical perspectives. EJNMMI Radiopharm Chem 2024;9:9

The decay of initially present ^221^Fr and ^213^Bi is shown with dotted lines, while the growth of ^221^Fr and ^213^Bi due to ^225^Ac decay is indicated with solid lines in Fig. 4. The half-life of ^221^Fr is 4.8 min, so after 30 min, the initially present ^221^Fr would have completely decayed. However, ^221^Fr generated from ^225^Ac decay will reach secular equilibrium by around 30 min. By adopting this method, RCP can be estimated, allowing the radiopharmaceutical to be released for patient use.

Bismuth-213 will reach equilibrium after 4–5 h, allowing gross gamma photon counting from ^221^Fr and ^213^Bi to provide a more accurate estimation of the RCP. Gamma counting can then be performed with a larger energy window to capture both emissions. The results from this measurement will provide an even more reliable estimation of the RCP.

### New ^18^F-prosthetic group, new possibilities

*By Ines Farinha Antunes*

Protein-based molecules against cancer biomarkers such as receptor tyrosine kinases (HER2, EGFR), integrin αvβ3, and interleukin-2 have been explored since they possess desirable properties such as high in vivo target affinity and selectivity. Additionally, in contrast to big proteins (antibodies), they have short biological half-lives that are highly compatible with a short-lived PET radionuclide such as fluorine-18. However, the incorporation of fluorine-18 into heat-sensitive proteins remains a challenge for radiochemists. Thus, proteins are often radiolabelled indirectly using ^18^F-prosthetic groups such as N-succinimidyl-4-[^18^F]fluorobenzoate ([^18^F]SFB) or N-[2-4-[^18^F]fluorobenzamido)-ethyl]maleimide ([^18^F]FBEM) that conjugate respectively to the lysine or the cysteine present in the protein. These indirect radiolabelling procedures are often complex (multi-step), time-consuming, and with relatively low RCY.

**Fig. 5:**
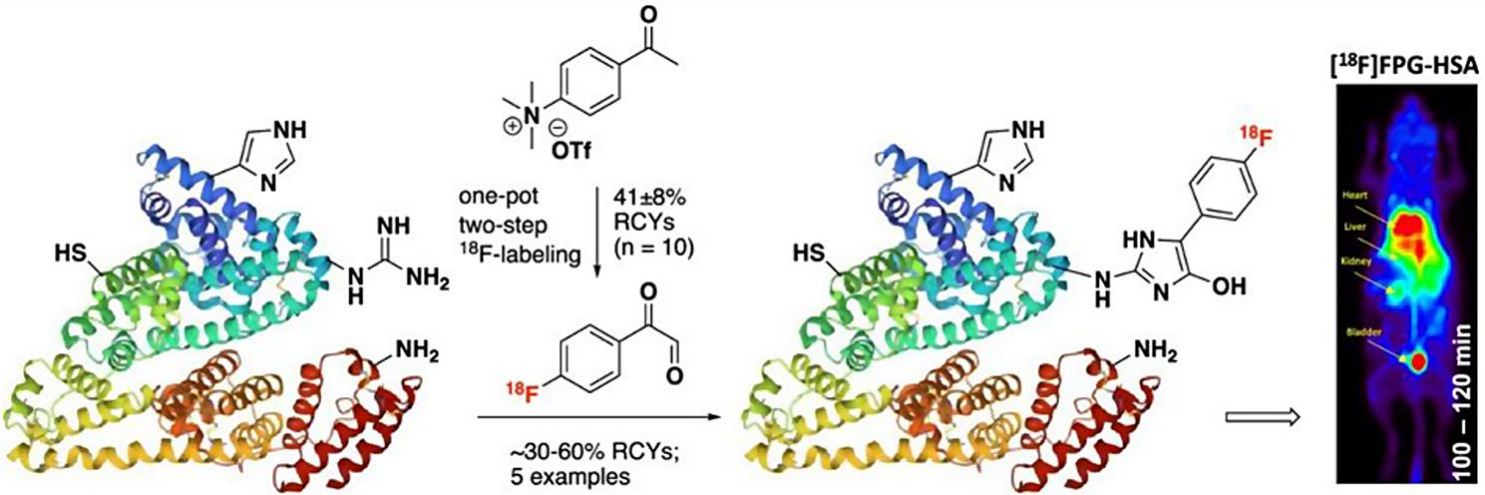
Synthesis of [^18^F]fluorophenylglyoxal and its conjugation to an arginine moiety of a protein. With permission from: Sadasivam P, Khanapur S, Harthimath SV, Ramasamy B, Cheng P, Feng CZ, Green D, Davis C, Goggi JL, Robins EG, Yan R. Arginine-Selective Bioconjugation Reagent for Effective ^18^F-labeling of Native Proteins. J Med Chem 2024;67:5064–5074

A new ^18^F-prosthetic group, [^18^F]fluorophenylglyoxal ([^18^F]FPG) has been developed that chemoselectively conjugates to arginine residues in proteins such as human serum albumin (HSA), ubiquitin, interleukin-2 (IL2) and interleukin-4 (IL4) with 30–60% activity yield (Sadasivam et al. 2024) (Fig. 5). [^18^F]FPG-HSA remains stable in vitro but also in vivo leading to less defluorination (2–6%) when compared to [^18^F]FBA-HSA (8–10%). The [^18^F]FPG-HSA indicated prolonged blood retention in mice proving that the addition of the radioactive prosthetic group did not alter the biological properties of HSA. The preservation of affinity in the ^18^F-radiolabelled cytokines (IL2 and IL4, respectively) was also confirmed in vitro. This study has proved that [^18^F]FPG is a suitable alternative to radiolabel small proteins as long as the protein is not pH-sensitive and its arginine groups are not involved directly in the binding to the receptor.

### Unlocking the potential of combined radionuclide and immunotherapy

*By Frederik Cleeren*

Immune checkpoint inhibitors (ICIs) often fail in treating advanced clear cell renal cell carcinoma (ccRCC), prompting the need for improved strategies. Therefore, the potential of combining targeted radionuclide therapy (TRT) with ICIs to enhance treatment efficacy was explored (Kleinendorst et al. 2024). Researchers used [^177^Lu]Lu-DOTA-hG250, targeting the overexpressed carbonic anhydrase IX (CAIX) in ccRCC, in combination with anti-PD-1 and anti-CTLA-4 ICIs.

The biodistribution of [^177^Lu]Lu-DOTA-hG250 was tested in mice with Renca-CAIX or CT26-CAIX tumors, which differ in T-cell infiltration and PD-L1 expression. Tumor-absorbed radiation doses were measured, and the efficacy of TRT with and without ICI was assessed by monitoring tumor growth and survival. Tumor tissues were analyzed before and after treatment using immunohistochemistry, flow cytometry, and RNA profiling. Results showed high uptake of [^177^Lu]Lu-DOTA-hG250 in both tumor models. Dose escalation studies in Renca-CAIX mice demonstrated that [^177^Lu]Lu-DOTA-hG250 had a dose-dependent anti-tumor effect. Combining a subtherapeutic TRT dose (4 MBq) with ICIs resulted in remarkable synergy and complete remissions. Similar results were observed in the CT26-CAIX model. Ex vivo analyses revealed DNA damage, increased T-cell infiltration, and modulated immune signaling pathways in the tumor microenvironment following combination treatment.

This proof-of-concept study demonstrates the outstanding therapeutic efficacy of combining subtherapeutic [^177^Lu]Lu-DOTA-hG250 with ICIs, leading to complete remissions and durable memory immune responses. These findings highlight the potential of combined TRT/ICI therapy for advanced ccRCC, providing insights for further mechanistic preclinical studies and paving the way for future clinical trials to maximize treatment benefits for patients.

### An old dog with new tricks: ^68^Ga-FAPI-04-PET for monitoring tumor responses to immunotherapy combinations in metastatic colorectal cancer

*By Zhaofei Liu*

Fibroblast activation protein (FAP)-targeted PET has been extensively investigated in clinical studies for the detection of various tumors and metastases. However, its utility in tracking tumor responses to inform precision therapies is limited. FAP is upregulated in cancer-associated fibroblasts, which produce immunosuppressive factors such as transforming growth factor-β (TGF-β), creating an immunosuppressive tumor microenvironment that can confer resistance to immunotherapy. It was shown that FAP-targeted PET with ^68^Ga-FAPI-04 can assess the effectiveness of combining TGF-β receptor (TGF-βR) inhibition with immune checkpoint blockade in metastatic colorectal cancer (Li et al. 2024). The trial involving 131 patients revealed that ^68^Ga-FAPI-04-PET provided complementary information to ^18^F-FDG-PET for cancer detection. Tumor tissue staining indicated that ^68^Ga-FAPI-04-PET could identify the immunosuppressive tumor microenvironment, which may act as a biomarker predicting tumor resistance to immunotherapy in patients. Following this, the authors turned to animal studies to assess the effectiveness of ^68^Ga-FAPI-04-PET in monitoring tumor responses to the combination immunotherapy of TGF-βR inhibition and a bispecific antibody targeting both PD-L1 and CTLA-4 in colorectal cancer metastasis models. The findings highlighted the sensitivity of ^68^Ga-FAPI-04-PET in monitoring tumor responses to the combined immunotherapy, potentially expanding the clinical role of FAP-targeted PET beyond tumor detection. Since FAP is also overexpressed in other disorders such as sclerosis, amyloidosis, and myocardial infarction, this study paves the way for FAP-targeted PET to guide personalized therapies for a broader spectrum of diseases.

### ^68^Ga-labelled peptide CK2 for PET imaging of NRP-1 expression

*By Beverley Ellis*

Neuropilin-1 (NRP-1) is a multifunctional glycoprotein which can be overexpressed in many cancer cells. It has been identified an effective target for the diagnosis and treatment of breast cancer, especially triple-negative breast cancer TNBC. NRP-1 targeting peptide radiotracers generally exhibit good biological properties but may have drawbacks of relatively poor stability, rapid clearance, and low permeability.

A novel NRP-1 peptide CK2 was designed and validated using in silico modelling and microscale thermophoresis assay with a view to improving the sensitivity and specificity detecting NRP-1 expression using PET imaging (Liu et al. 2024). [^68^Ga]Ga-NOTA-PEG_4_-CK2 was synthesized with a high radiochemical yield and showed high in vitro stability. In vitro and in vivo studies were undertaken to assess the specificity and sensitivity of this radiotracer.

In vitro cellular uptake assay demonstrated that the radiotracer specifically binds to NRP-1 positive cancer cells (MDA-MB-231) rather than NRP-1 negative cancer cells (NCI-H1299). MicroPET imaging showed a significant accumulation of [^68^Ga]Ga-NOTA-PEG_4_-CK2 in MDA-MB-231 tumors compared with NCI-HI299 tumors. High specificity of [^68^Ga]Ga-NOTA-PEG_4_-CK2 for NRP-1 expression was also confirmed by ex vivo biodistribution, autoradiography, western blot and immunofluorescence staining. The expression of NRP-1 in MDA-MB-231 tumors and cells was shown to be down-regulated by SB-203,580 treatment which could be sensitively monitored by [^68^Ga]Ga-NOTA-PEG_4_-CK2. The authors conclude that [^68^Ga]Ga-NOTA-PEG_4_-CK2 is a promising new radiotracer for the detection NRP-1 expression and could provide useful information for evaluating the prognosis of breast cancer.

### Peptidoglycan-targeting radiotracers for bacteria-specific imaging

*By Maryke Kahts*

Infection and antimicrobial resistance have been major topics of discussion in recent years, with continuous global efforts focused on preserving antibiotic efficacy. Among these efforts is the early identification and localization of the causative bacterial pathogen to initialize the most appropriate treatment regimen as soon as possible. All existing infection detection methods have their respective limitations. Current nuclear medicine imaging approaches utilize radiopharmaceuticals that lack specificity for infection, e.g. [^18^F]fluorodeoxyglucose and radiolabeled leukocytes. Bacteria-specific radiotracers have therefore emerged as a hot topic. The peptidoglycan layer in the bacterial cell wall offers the potential to develop radiotracers that can accurately and specifically detect infection and possibly distinguish between Gram-negative and Gram-positive bacterial strains.

**Fig. 6:**
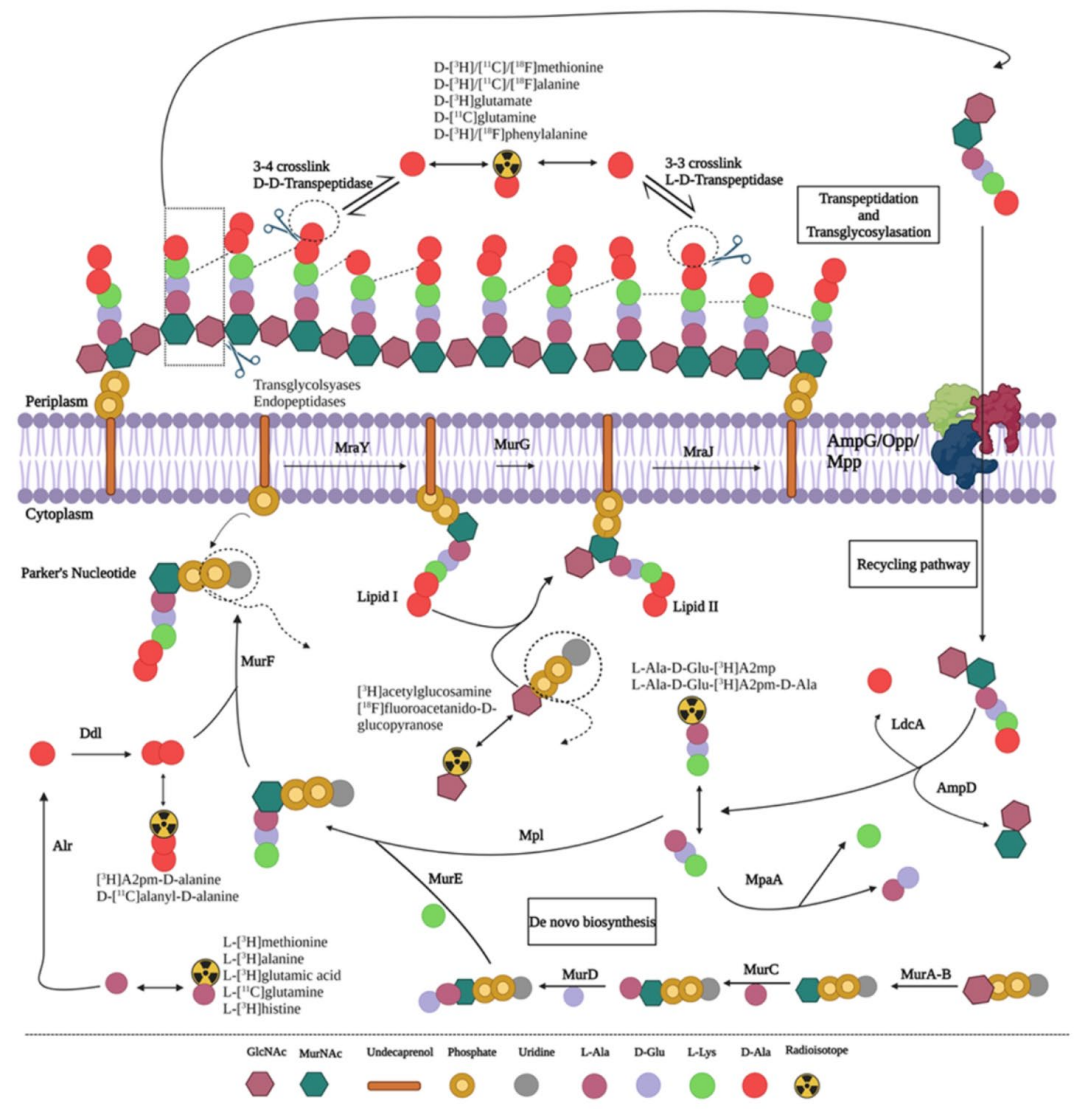
Proposed pathways of peptidoplycan biosynthesis and potential precursors for designing radiotracers or probes from imaging. With permission under licence CC BY 4.0 from Koatale P, Welling M, Ndlovu H, Kgatle M, Mdanda S, Mdlophane A, Okem A, Takyi-Williams J, Sathekge M, Ebenhan T. Insights into Peptidoglycan-Targeting Radiotracers for Imaging Bacterial Infections: Updates, Challenges, and Future Perspectives. ACS Infectious Diseases 2024;10:270–286

Recent investigations on radiotracers targeting peptidoglycan biosynthesis were summarized (Koatale et al. 2024) (Fig. 6). Some promising radiotracers identified are amino acid-based tracers, e.g. D-[^11^C]methionine, D-[^11^C]alanine and D-[^11^C]glutamine, D-amino acid dipeptide-based probes, e.g. D-[^11^C]alanyl-D-alanine, Park’s nucleotide-based probes, e.g. UDP-MurNAc-[^11^C]pentapeptide, lipid-based probes, e.g. [^11^C]GlcNAc-labeled lipid II, glycan core-based probes, e.g. [^3^H]acetylglucosamine and [^18^F]fluoroacetamido-D-glucopyranose, and oligopeptide-based probes, e.g. L-ala-D-glu-[^3^H]A2pm. Specifically, D-[^11^C]methionine illustrated promising clinical application in suspected prosthetic joint infections and D-[^11^C]alanine in spinal infections, pneumonia and antibiotic-resistant infections.

The limitations in developing peptidoglycan biosynthesis-targeting radiotracers were also highlighted, including complex chemical synthesis, the challenging radiochemistry of fluorine-18 and carbon-11, cyclotron production of these radionuclides, and the associated cost and sophistication of the required infrastructure (Koatale et al. 2024). The authors concluded that technological innovation in biology, chemistry and radiochemistry, with a multidisciplinary approach is needed for clinically translatable research in this field.

### Latest trends on radiopharmaceuticals for bacterial infection imaging

*By Fany Pricile Ekoume*

Appropriate therapies with a high rate of antimicrobial resistance is a great concern in African hospitals (Ngogang et al. 2024). The same trend is observed worldwide with the prediction that the major cause of death by 2050 would be drug resistant infections. To overcome the threat posed by antimicrobial resistance to the health care system, factors including personalized medicine utilizing radionuclide imaging with fast and accurate diagnosis of infections and reliable identification of intractable and resistant infection are crucial for the application of antibiotic stewardship and the overuse of broad-spectrum antibiotics (Kahts et al. 2024). Nowadays, the development and investigation of new radiopharmaceuticals based on differentiation of infection from sterile inflammation as well as the imaging of specific infectious microbes’ processes using both PET and SPECT products could help to approach the issue in a holistic manner.

A recent review of the most prominent discoveries related to radiopharmaceuticals development and characterization of bacterial infection imaging agents includes radiolabelled bacterial siderophores to target *E. coli*-induced infections. More so, fungal siderophores were studied using small animal PET imaging in a mouse model of infection and achieving RCP higher than 95% (Krasulova et al. 2024). Moreover, antimicrobial peptides labelled with ^64^Cu, ^68^Ga, and ^99m^Tc was documented as well as several radiolabelled antibiotics investigations performed. The preferred tracer would be dependent on the clinical scenario, with consideration of its ability firstly, to detect all or most pathogenic microorganisms, secondly to distinguish infection from other processes, lastly to play a role to identify specific bacterial strains for the selection of the most appropriate and effective antimicrobial therapy. Selected radiopharmaceuticals were highlighted with regard to on one hand, their promising first-in-human application, on the other hand, significant results from well-structured conceptional clinical investigations in small patient populations, and ultimately, on evidence from early clinical trials.

The authors conclude that the key to reliable evaluation of the true potential of the newly proposed infection imaging agents include a more structured and harmonized preclinical setting as well as well-designed clinical investigations (Kahts et al. 2024).

### New options for treating patients with NETs

*By Ivis F Chaple*

Recent advances in Somatostatin Receptor (SSTR)-targeted diagnostic and therapeutic radiopharmaceuticals have resulted in clinical indications for patients with SSTR positive Neuroendocrine Tumors (NETs). The importance of continued research and development for NET imaging and targeted therapy was recently demonstrated (Gomes et al. 2024). This article highlights the need to perform dosimetry computations to generate estimated organ absorbed dose for all radiopharmaceuticals, to ascertain safety profiles and feasibility for clinical translation, particularly when options already exist in the clinical space. Performing dosimetry computations for radiopharmaceuticals prior to pursuing clinical translation efforts could lead to emphasise on studying modifications of peptides for improved receptor specificity and pharmacokinetic profiles. The study further improves the landscape for radiopharmaceuticals targeting SSTR + NETs by providing absorbed dose estimates for [^43/44/44m^Sc]Sc-DOTA-TATE compared to the Food and Drug Administration (FDA) approved [^68^Ga]Ga-DOTA-TATE and ^111^In-Octreotide. It was concluded that their data indicate a reasonably higher absorbed dose per injected activity of [^43/44/44m^Sc]Sc-DOTA-TATE in organs at risk as compared to the dose delivered by [^68^Ga]Ga-DOTA-TATE. However, this assumes that both radiopeptides distribute equally in the body. The calculated doses are within a reasonable order of magnitude of other clinically used radionuclides, indicating feasibility of employing [^43/44/44m^Sc]Sc-DOTA-TATE for PET imaging of SSTR + NETs. Implementation of [^43/44/44m^Sc]Sc-DOTA-TATE as diagnostic imaging options for patients could prove beneficial due to the more similar chemical characteristics of Sc (compared to Ga) and Lu, of which ^177^Lu is currently employed for targeted therapy of SSTR + NETs. The suggestion of utilizing radioscandium provides an important discussion on research and development in the areas of exotic radionuclide implementation, as they could provide enhanced radiochemistry capabilities and, ultimately, serve as better pairs for some therapeutic counterparts.

### Towards a microfluidic synthesis of PET radiopharmaceuticals

*By Emerson Bernardes*

Outside the academic sphere, innovative concepts often materialize through the establishment of startups. These new companies are founded by one or more entrepreneurs who aim to develop a product or service they perceive to have market demand. However, the most frequent cause of startup failure is the absence of a market need for the offered product or service, which indicates the solution provided is not compelling enough for customers to invest in – simple as that.

In academia, the development of integrated and automated microfluidic platforms for radiotracer production is ongoing, focusing on new methods to avoid azeotropic drying and creating automated systems that interface with existing production methods and comply with GMP standards (Ovdiichuk et al. 2024). However, the expected demand for these technologies in the radiopharmaceutical market has not yet materialized as anticipated. Experts in the field argue that: (1) implementing microfluidic systems for radiopharmaceutical production is crucial for enabling personalized care through dose-on-demand radiotracer production; and (2) the high cost of radiotracer synthesis and the necessity for highly trained operators to manage commercial equipment are major factors preventing the introduction of new PET radiopharmaceuticals and the reason why [^18^F]FDG remains the predominant choice, accounting for over 95% of procedures (McVeigh and Bellan 2024). Therefore, shifting from multi-dose to single-dose batches would enable hospitals and nuclear medicine centers to reduce costs and manufacture other non-commercialized PET radiopharmaceuticals.

Reflecting on over two decades of microfluidic platform development, the integration of this technology into the radiopharmaceutical market seems increasingly inevitable. Perhaps, the key question is not whether microfluidics will be adopted, but in which segment of the radiopharmaceutical market it will have the most significant impact. The new microfluidic platforms and methods are well-suited for: (1) small-scale commercial manufacturing of numerous new radiopharmaceuticals currently under evaluation or soon to be evaluated in clinical trials; (2) production of short-lived PET radionuclides, such as ^15^O (t_1/2_ = 2 min), ^13^N (t_1/2_ = 10 min), and ^11^C-based radiopharmaceuticals, for research purposes in academic institutions; (3) supporting the research and development of novel radiopharmaceuticals in non-clinical stage; and (4) purification of radionuclides.

At present, there is no global movement towards dose-on-demand in the radiopharmaceutical market, and the cost of radiopharmaceutical production is not a significant obstacle to the widespread adoption of new radiopharmaceuticals beyond ^18^F-FDG. A key reason why new tracers do not achieve widespread use is not necessarily the production cost, but rather the absence of clear, patient-relevant clinical outcomes. Ultimately, if the benefits for patients are not compelling, there will be no demand for the new tracers.

If there is no market demand for a product or service, startups typically fail and disappear, either before they exhaust their funds or soon after, with financial depletion being the second most common reason for startup failures. Unfortunately, in academia, once misleading information and unrealistic expectations about the widespread adoption of microfluidic platforms for radiopharmaceutical production are published, they tend to persist without contributing to the field’s development.

### Peptide binder to glypican-3 as a theranostic agent for hepatocellular carcinoma

*By Martin Behe*

The authors describe the evaluation of a radiolabeled bicyclic peptide targeting Glypican-3 (Lin et al. 2024). Glypican-3 is involved in embryonic development with a low level of expression in healthy tissue after birth. But it is highly expressed in tumors like hepatocellular carcinoma (HCC), lung adenocarcinoma, squamous cell carcinoma, embryonal tumors, testicular germ cell tumors, and liposarcoma. Therefore, it is a highly interesting target for radioligand therapy.

**Fig. 7:**
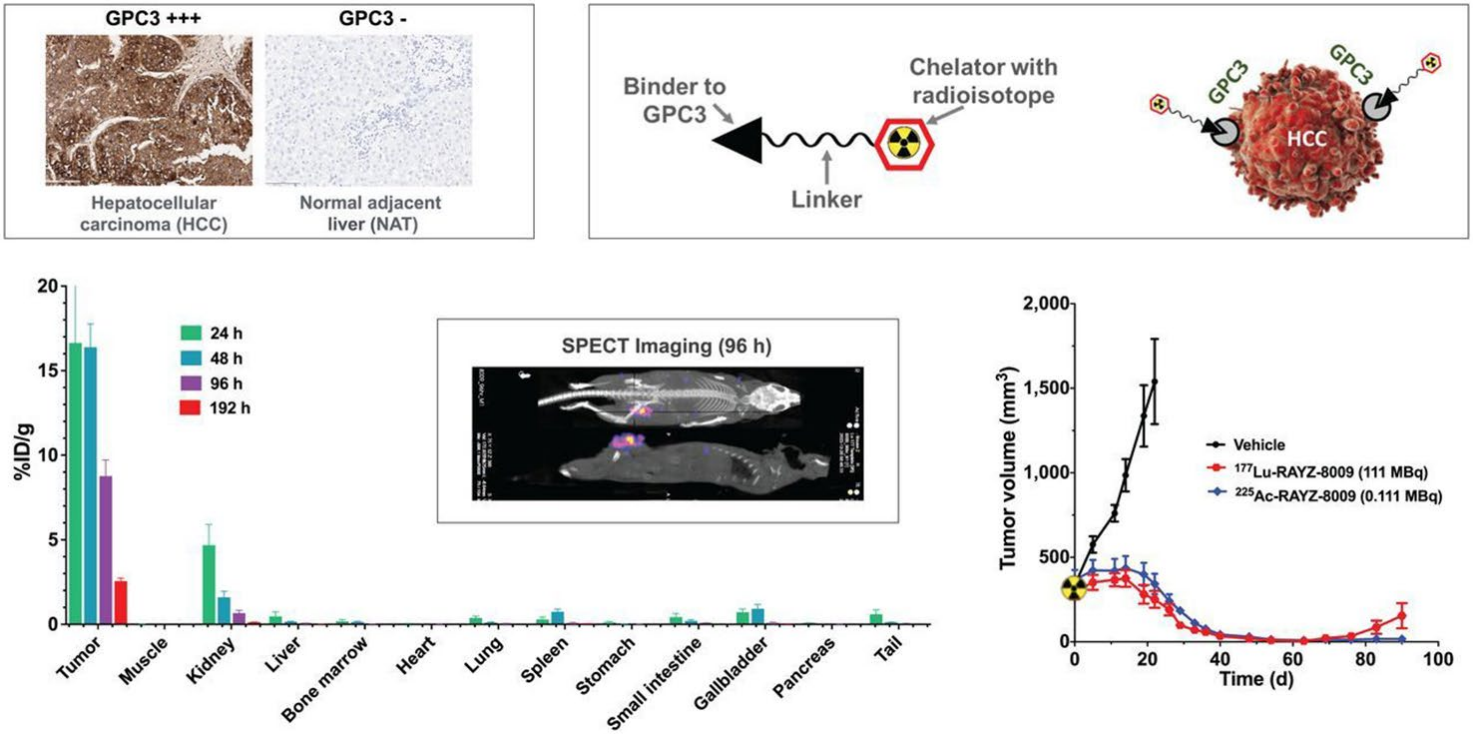
Graphical abstract of the biological evaluation of the peptide binder to glypican-3. This research was originally published in *JNM*. Fanching Lin, Renee Clift, Takeru Ehara, Hayato Yanagida, Steven Horton, Alain Noncovich, Matt Guest, Daniel Kim, Katrina Salvador, Samantha Richardson, Terra Miller, Guangzhou Han, Abhijit Bhat, Kenneth Song and Gary Li. Peptide Binder to Glypican-3 as a Theranostic Agent for Hepatocellular Carcinoma. J Nucl Med. 2024;4:586–592. © SNMMI

The authors evaluated a DOTA coupled bicycle peptide (RAYZ-8009) which was identified with a peptide library screen with actinium-225 and lutetium-177 (Fig. 7). A saturation binding assay with [^177^Lu]Lu-RAYZ-8009 revealed a dissociation constant of 10.8 nM. Inhibitory binding assays showed no significant differences between different isotopes (lanthanum were used as a surrogate for actinium).

The biodistribution study in mice bearing a tumor derived from HepG2 (human HCC cell line) showed a high uptake in the receptor positive tumor with a slow release (lutetium-177, 1 h: 25% i.a./g; 96 h: 8.8% i.a./g). The kidneys are the only organs with significant uptake but they exhibit a fast clearance (1 h: 20% i.a./g; 96 h: 0.67% i.a./g). Therapeutic studies with actinium-225 and lutetium-177 radiolabeled RAYZ-8009 with two different HCC tumors xenografts showed a significant reduction of tumor growth with even a complete remission with 111 kBq [^225^Ac]Ac-RAYZ-8009 in Hep3B xenografts.

Overall this is a very nice example of a radioconjugate derived from a library screening with a highly promising preclinical evaluation and high potential in clinical applications. A weakness of the paper is that the structural information is missing which are highly necessary to completely understand the results from a scientific point of view.

### Novel combination strategy for ^177^Lu-labelling of antibody under mild condition

*By Ya-Yao Huang*

To develop an approach with both advantages of radiometal-chelation under mild conditions and rapid antibody-conjugation is important for radioimmunotherapy. The novel nonadecadentate bispidine (Bisp, 3,7-diazabicyclo[3.3.1]nonane) has been shown the ability to achieve coordinatively saturated lanthanide (III)-protein complexes with high thermodynamic and kinetic stability (Cieslik et al. 2022). On the other hand, one-pot photochemical antibody-conjugation methods have successfully been applied in ^68^Ga-labeled antibodies with the chelator bearing a photoactivatable ArN_3_ group (Patra et al. 2019; Fay et al. 2019). Two approaches above were recently combined to conduct the [^177^Lu]Lu-Bisp-ArN3 chelation at 40℃ for 10 min and following photo-conjugation of Trastuzumab at 23 °C/395 nm for 15 min, and successfully achieved final product of [^177^Lu]Lu-Bisp-Trastuzumab with 12–14% of decay-corrected radiochemical yield and > 90% of radiochemical purity (Cieslik et al. 2024). However, low radioactivity concentration may cause the lower tumor uptake compared to ^89^Zr-labelled Trastuzumab (i.e., 30% vs. 66%). In particular, the stability of [^177^Lu]Lu-Bisp-Trastuzumab in human serum may be concerned for the clinical use, because of the degradation after 24 h even under low radioactivity concentration.

The in vivo stability depending on the radioactivity concentration has been defined as a key parameter during the development of ^177^Lu-labeled radiopharmaceuticals, especially when high levels of [^177^Lu]LuCl_3_ activity (> 1GBq) is commonly used in a clinical setting. Consequently, the feasibility of fully automated radiosynthesis of [^177^Lu]Lu-Bisp-Trastuzumab using the custom-built ALISI device (Klingler et al. 2022) and the role of different radiolysis quenchers both warrant further investigation (Larenkov et al. 2023; Schmitl et al. 2023).

### Cholecystokinin-2 receptor targeting with theranostic potential

*By Renata Mikolajczak*

Recently published as the EJNMMI image of the month, cholecystokinin-2 receptor (CCK2R) targeting by [^68^Ga]Ga-DOTA-MGS5 PET/CT in a patient with extensive disease small cell lung cancer (Di Santo et al. 2024) provides an excellent opportunity to highlight the role gastrin/cholecystokinin-2 receptors as molecular targets.

CCK2 receptors are expressed at high incidence in medullary thyroid carcinomas (MTC, > 90%), small cell lung cancers (> 50%), astrocytomas (> 60%), insulinomas, stromal ovarian cancers, gastrointestinal stromal tumors, and more than 20% of gastroenteropancreatic tumors (Reubi et al. 1997). Although the CCK2R-targeting peptide probes are based on the natural ligands cholecystokinin and gastrin, the high kidney uptake and the limited enzymatic stability of these linear peptide analogues in vivo created a challenge for researchers. DOTA-MGS5 (DOTA-d-Glu-Ala-Tyr-Gly-Trp-(N-Me)Nle-Asp-1-Nal-NH_2_) proposed by the Innsbruck group in 2019 was among the newest minigastrin analogues with an optimized targeting profile (Klingler et al. 2019). In preclinical studies, it demonstrated a high and persistent tumor uptake and favourable tumor-to-background activity ratios, including kidneys.

To date, only a few CCK2 receptor-targeting radioligands have been used in a clinic with a focus on MTC patients. [^68^Ga]Ga-DOTA-MGS5 showed high uptake in all FDG-avid lesions and identified additional abnormal foci in the patient with advanced small cell lung cancer, suggesting disease progression (Di Santo et al. 2024). Since DOTA-MGS5 can also be labelled with ^177^Lu, the CCK2 receptor targeting can be considered for therapy. These promising findings encourage further clinical studies oriented at the CCK2 receptor targeting of advanced-stage CCK2R-expressing malignancies.

### Revolutionizing radiochemistry: high-throughput experimental approach for efficient cu-mediated ¹⁸F-radiofluorination

*By Shozo Furumoto*

**Fig. 8:**
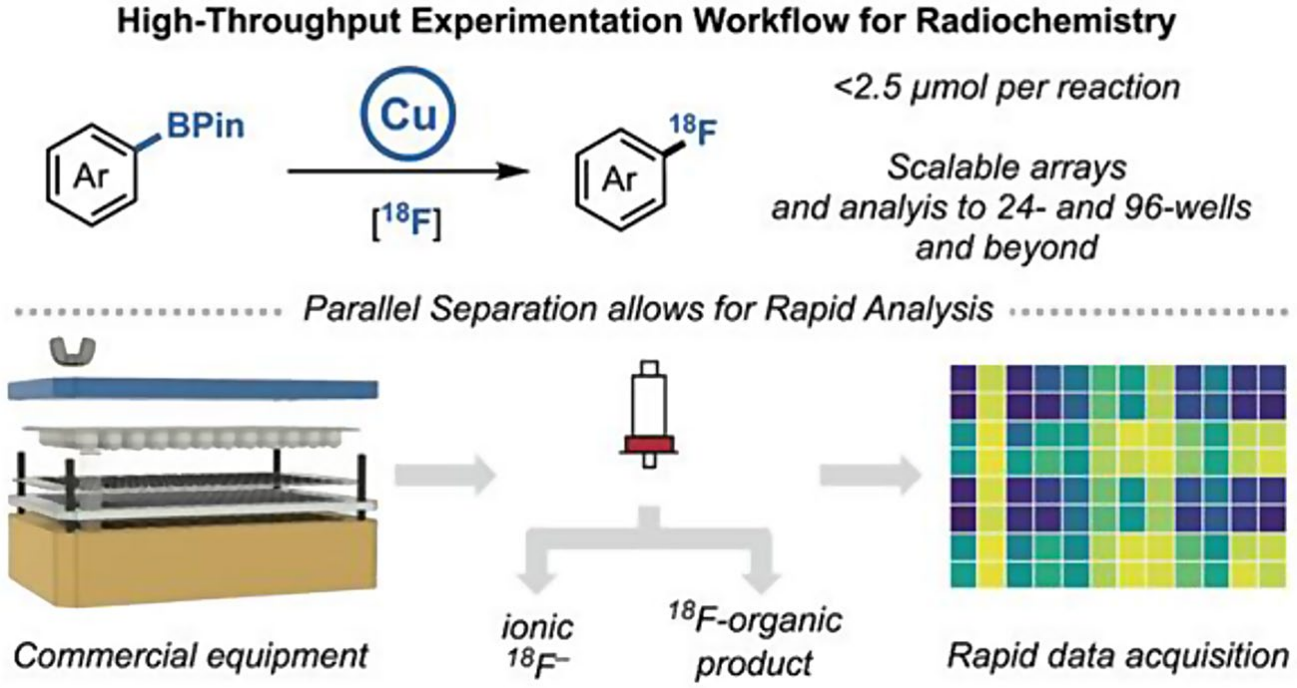
Schematic overview of the High Throughput Experiments by performing ^18^F-fluorinations in multiwell plates, with subsequent separation and analysis. *With permission from* Webb EW, Cheng K, Winton WP, Klein BJC, Bowden GD, Horikawa M, Liu SW, Wright JS, Verhoog S, Kalyani D, Wismer M, Krska SW, Sanford MS, Scott PJH. Development of High-Throughput Experimentation Approaches for Rapid Radiochemical Exploration. J Am Chem Soc 2024;146:10581–10590

High-throughput experimentation (HTE) has revolutionized drug discovery by enabling rapid identification of promising compounds and efficient optimization of reaction conditions. However, its application to radiolabeled drug development, particularly for PET radiopharmaceuticals, has been underutilized. Recently, the groups of Melanie S. Sanford and Peter J. H. Scott introduced a novel HTE platform for copper-mediated radiofluorination (CMRF) of aryl boronates using ¹⁸F (Webb et al. 2024) (Fig. 8). In this study, a total of 16 different reaction conditions for 31 compounds were investigated to determine optimal labeling conditions. By integrating commercial HTE instrumentation with advanced analytical techniques-including PET scanners, gamma counters, and autoradiography (ARG)- research demonstrates significant time and cost savings while increasing the efficiency of reaction optimization. The group compared radiochemical conversion (RCC) values from PET scanners, gamma counters, and ARG with those from conventional radio-TLC, a widely used method for calculating RCC. They found that PET scanners and gamma counters showed strong correlations with RCC values from radio-TLC, confirming the reliability of these high-throughput methods. The results of this study underscore the potential for broader adoption of HTE in radiochemistry, not only for ¹⁸F but also for other radionuclides, which could accelerate the development of radiopharmaceuticals. The study also points out limitations, such as challenges in product identification and the need for further automation. However, the study provides a roadmap for future advances in high-throughput radiochemistry. Overall, this research represents a significant advance, offering both practical solutions and new directions for optimizing complex radiolabeling reactions in a more efficient and scalable manner.

### In vivo stable ^211^At-labeled prostate-specific membrane antigen-targeted tracer using a neopentyl glycol structure

*By Amal Elrafaei*

Prostate cancer is common globally (Sung et al. 2021), with metastatic castration-resistant prostate cancer (mCRPC) being hard to treat. PSMA, overexpressed in mCRPC, is a key target for targeted alpha therapy (TAT). A novel TAT by designing a stable PSMA-targeted tracer labeled with the alpha-emitting radiohalogen astatine-211 is presented, using a neopentyl glycol (NpG) structure which is being stable against dehalogenation (Suzuki et al. 2024).

The biodistribution of ^211^At-labeled PSMA derivatives was studied in normal and tumor-bearing mice. Results indicate [^211^At]At-NpG-D-PSMA shows promise as a new TAT for mCRPC due to its in vivo stability and favourable biodistribution. Comparisons with derivatives suggest that modifications, such as using a D-form glutamic acid linker, enhance in vivo stability and reduce unwanted metabolization. The findings suggest that [^211^At]At-NpG-D-PSMA could be a promising candidate for further mCRPC treatment development.

The study confirms that PSMA ligand derivatives with D-form amino acids, show higher tumor accumulation than those with L-form amino acids, consistent with previous findings (Weineisen et al. 2014). The configuration of the glutamic acid linker affects renal retention, with D-form linkers having shorter retention times in the kidney compared to L-form linkers.

This study observed that the NpG structure effectively retains astatine-211 in vivo, as shown by low accumulation in the stomach and thyroid. [^211^At]At-NpG-D-PSMA maintained stable tumor radioactivity with decreased renal radioactivity over time, making it a promising candidate for TAT in mCRPC due to its favourable biodistribution, stability, and tumor accumulation.

### The critical amount of activity in the target tissue for targeted radioligand therapy (TRT): is it achievable if the binding-to-ligation transition concept is introduced using the click SuFEx reaction?

*By Klaus Kopka*

**Fig. 9:**
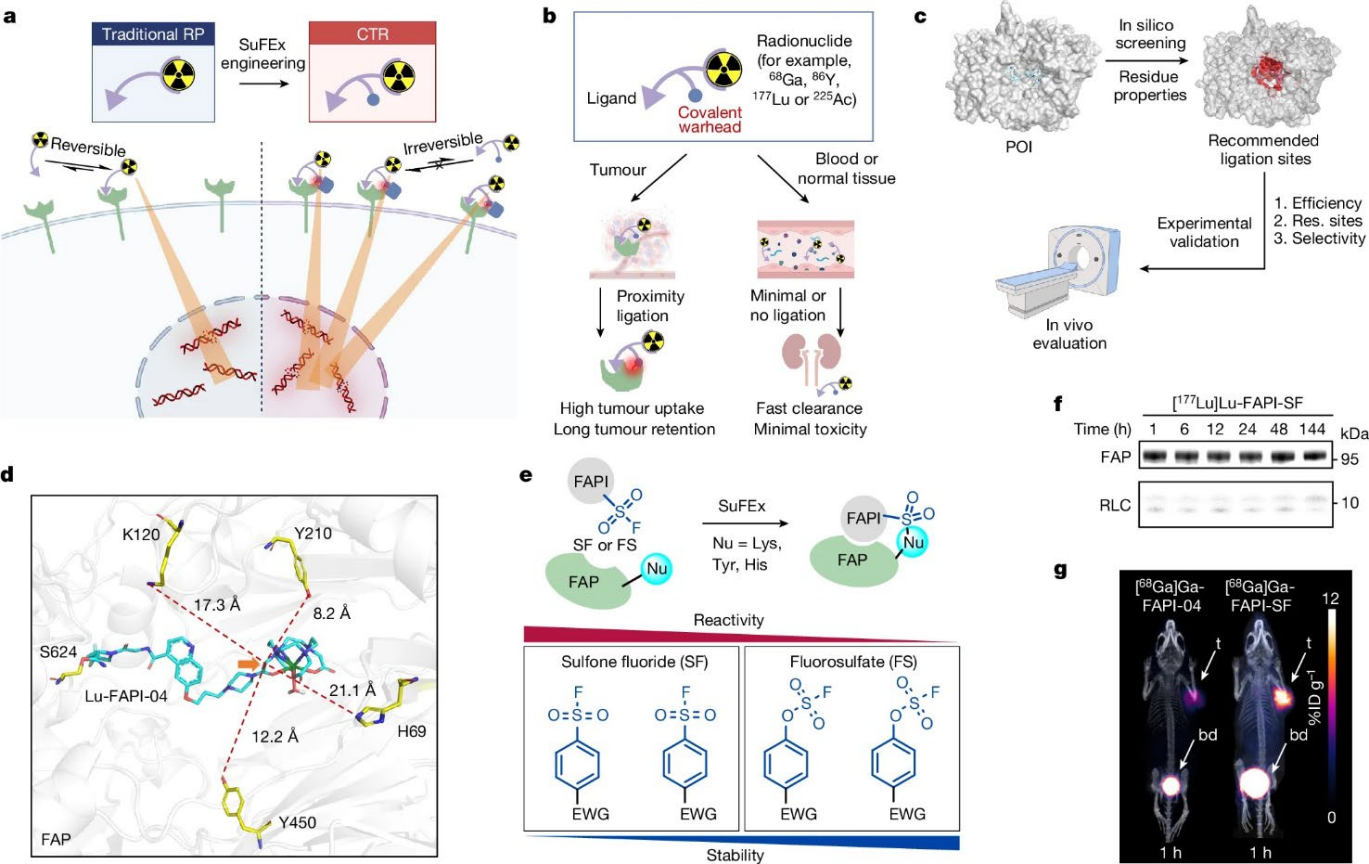
Development of a CTR by SuFEx engineering and successful proof of concept in tumor-bearing mice. **a**, Schematic representation of the potential benefits of CTRs over traditional radiopharmaceuticals (RPs) for TRT. **b**, Ideal working model of a CTR. **c**, Overall procedure for CTR development. **d**, Molecular docking of Lu-FAPI-04 (cyan) against FAP (grey; Protein Data Bank (PDB): 1Z68) showing the selected possible ligation residues. The carbon selected for SuFEx engineering in this work is indicated with an orange arrow. **e**, Scheme of proximity-enabled SuFEx ligation on FAP. The SuFEx covalent warheads screened in this work and the relationships between structure, activity and stability are summarized. EDG, electron-donating group; EWG, electron-withdrawing group; Nu, nucleophile at side chain. **f**, Analysis of FAPI-SF binding to FAP in vitro. Purified human FAP (3 µM) in PBS buffer (pH 7.4) was incubated with [^177^Lu]Lu-FAPI-SF (0.15 µM) at 37 °C. The upper bands indicate that FAPI-SF is irreversibly bound to FAP. **g**, PET/CT images of the same HT-1080-FAP tumor-bearing mouse intravenously injected with [^68^Ga]Ga-FAPI-04 and [^68^Ga]Ga-FAPI-SF, respectively, at a 12-h interval. bd, bladder; ID, injected dose; t, tumor. Data are representative of three independent experiments (f). RLC, radionuclide-ligand conjugate. The illustrations in a–c were created with BioRender. Reproduction permitted *via* RightsLink from Cui XY, Li Z, Kong Z, Liu Y, Meng H, Wen Z, Wang C, Chen J, Xu M, Li Y, Gao J, Zhu W, Hao Z, Huo L, Liu S, Yang Z, Liu Z. Covalent targeted radioligands potentiate radionuclide therapy. Nature 2024;630:206–213

This research article (Cui et al. 2024) aimed at increasing the amount as well as the retention of targeted radioligands, giving the example of a FAP targeting tracer, by additional implementation of a sulfur (VI) fluoride exchange (SuFEx) chemistry-based linker into the radioligand. This clever idea is based on the fact that the engineered radioligand binds firstly to the tumor-specific protein, and subsequently undergoes a binding-to-ligation transition in proximity of the protein’s binding pocket thereby conjugating to a tyrosine residue through the desired ‘click’ SuFEx reaction. The authors name this concept a covalent targeted radioligand (CTR) approach (Fig. 9). Indeed, they observed that this strategy, using aryl-sulfone fluoride (FS) as covalent warhead, in a FAP inhibitor (FAPI) triggered more than 80% covalent binding to FAP. At the same time no dissociation was observed for six days. In HT-1080-FAP cell-line-derived xenograft (CDX) mouse models, the SuFEx-engineered and ^68^Ga-labelled FAPI (FAPI-mFS) showed 257% greater tumor uptake than the original [^68^Ga]Ga-FAPI-04, and increased 13-fold tumor retention. The clearance of tracer-associated activity from normal tissues/organs was maintained. The authors even transferred their idea into the clinical scenario. In their pilot PET/CT imaging study, this covalent targeted radioligand approach identified more tumor lesions in patients suffering from medullary thyroid carcinoma (MTC) than other methods. The authors conclude that the SuFEx-engineered FAPI also can be transferred to targeted β- and α-radionuclide therapy. Moreover, the authors examined a SuFEx-engineered PSMA-ligand based on PSMA-617 (Pluvicto) that showed enhanced LNCaP-tumor uptake as well as increased therapeutic efficacy. All in all, the binding-to-ligation transition using CTRs, bearing SuFEx chemistry-based linkers, can potentially be adapted also to other cancer targets.

## Conclusions

Trends in radiochemistry and radiopharmacy are highlighted. Hot topics cover the entire scope of EJNMMI Radiopharmacy and Chemistry, demonstrating the progress in the research field in many aspects.

## Data Availability

Datasets mentioned in this article can be found in the cited articles.

## Abbreviations

CTR: Covalent targeted radioligand
EGFR: Epidermal growth factor receptor
FAP: Fibroblast activation protein
FDG 2-[^18^F]: Fluoro-2-deoxyglucose
GMP: Good manufacturing practice
HAP: Hydroxy apatite
HCC: Hepatocellular carcinoma
HAS: Human serum albumin
THE: High throughput experimentation
ICD: Immunogenic cell death
ICI: Immune checkpoint inhibitor
IL: Interleukin
ISV: In situ vaccination
mCRPC: Metastatic castration-resistant prostate cancer
MTC: Medullary thyroid carcinoma
NET: Neuroendocrine tumor
PET: Positron emission tomography
PSMA: Prostate-specific membrane antigen
RCC: Radiochemical conversion
RCP: Radiochemical purity
RCY: Radiochemical yield
RGD: Arginyl glycyl aspartic acid
SPECT: Single photon emission computed tomography
SSTR: Somatostatin receptor
SuFEx: Sulphur fluoride exchange
TAT: Targeted alpha therapy
TGF: Transforming growth factor
TLC: Thin layer chromatography
